# Antibiotic definitive treatment in ventilator associated pneumonia caused by AmpC-producing Enterobacterales in critically ill patients: a prospective multicenter observational study

**DOI:** 10.1186/s13054-024-04820-7

**Published:** 2024-02-05

**Authors:** Matthieu Petit, Frank Bidar, Quentin Fosse, Lucie Lefevre, Marine Paul, Tomas Urbina, Paul Masi, Florent Bavozet, Jérémie Lemarié, Etienne de Montmollin, Chloé Andriamifidy-Berti, Julien Dessajan, Benjamin Zuber, Lara Zafrani, Edwige Peju, Paris Meng, Liliane Charrier, Loic Le Guennec, Marie Simon, Charles-Edouard Luyt, Luc Haudebourg, Guillaume Geri

**Affiliations:** 1 Medical Intensive Care Unit, Ambroise Paré Hospital, APHP, UMR 1018, CESP Villejuif, 9, Avenue Charles de Gaulle, Boulogne-Billancourt, France; 2 Anesthesia and Critical Care Medicine Department, Hospices Civils de Lyon, Edouard Herriot Hospital, Lyon, France; 3 AP-HP, Service de Médecine Intensive-Réanimation, Hôpital de Bicêtre, DMU CORREVE, Inserm UMR S_999, FHU SEPSIS, Groupe de Recherche Clinique CARMAS, Université Paris-Saclay, Le Kremlin-Bicêtre, France; 4 Médecine Intensive Réanimation, Institut de Cardiologie, Groupe Hospitalier Pitié-Salpêtrière, Assistance Publique-Hôpitaux de Paris, Sorbonne-Université, Hôpital Pitié-Salpêtrière, Paris, France; 5 Intensive Care Unit, Centre Hospitalier de Versailles-Site André Mignot, Le Chesnay, France; 6 Service de Médecine Intensive Réanimation, Hôpital Saint-Antoine, Assistance Publique des Hôpitaux de Paris, Paris, France; 7 AP-HP, Hôpitaux Universitaires Henri-Mondor, Service de Médecine Intensive Réanimation, 94010 Créteil, France; 8 CH de Dreux, Médecine Intensive Réanimation, Dreux, France; 9 Service de Médecine Intensive Réanimation, Centre Hospitalier Universitaire de Nantes, Centre Hospitalier Universitaire Hôtel-Dieu, Nantes, France; 10 INSERM UMR 1137, 75018, Department of Intensive Care Medicine, APHP, Bichat-Claude Bernard University Hospital, Université Paris Cité, 75018 Paris, France; 11 Médecine Intensive - Réanimation, Centre Hospitalier de Poissy - Saint Germain en Laye, Poissy, France; 12 Service de Médecine Intensive-Réanimation, Hôpital Tenon, Assistance Publique-Hôpitaux de Paris, Paris, France; 13 Intensive Care Unit, Hôpital Foch, 92150 Suresnes, France; 14 Medical Intensive Care Unit, Saint-Louis Hospital, AP-HP, University of Paris Cité, Paris, France; 15 Medical Intensive Care Unit, Hôpital Cochin, Assistance Publique-Hôpitaux de Paris AP-HP Centre, Université Paris Cité, Paris, France; 16 Service de Médecine Intensive Réanimation, CHI Robert Ballanger, Aulnay-sous-Bois, France; 17 Service de Réanimation, Centre Hospitalier du Cotentin, Cherbourg, France; 18 Médecine Intensive Réanimation Neurologique, Hôpital de la Pitié-Salpêtrière - APHP, Sorbonne Université, Paris, France; 19 Médecine Intensive Et Réanimation, CHU Edouard Herriot, Lyon, France; 20 Service de Médecine Intensive Réanimation, Institut de Cardiologie, ICAN, Groupe Hospitalier Pitié-Salpêtrière, Assistance Publique-Hôpitaux de Paris, Sorbonne-Université, Hôpital Pitié-Salpêtrière, Paris, France; 21 Service de Pneumologie et Réanimation Médicale du Département R3S, Groupe Hospitalier Pitié-Salpêtrière Charles Foix, AP-HP, Paris, France; 22 Medical and Surgical Intensive Care Unit, Ambroise Paré Clinic, Neuilly-sur-Seine, France

**Keywords:** AmpC β-lactamase, Enterobacterales, Ventilator associated pneumonia, Third generation cephalosporin, Intensive care unit

## Abstract

**Background:**

Ventilator associated pneumonia (VAP) due to wild-type AmpC-producing Enterobacterales (wtAE) is frequent in intensive care unit (ICU) patients. Despite a low level of evidence, definitive antimicrobial therapy (AMT) with third generation cephalosporins (3GCs) or piperacillin is discouraged.

**Methods:**

Observational prospective study including consecutive wtAE VAP patients in 20 French ICUs. The primary objective was to assess the association of the choice of definitive AMT, i.e. piperacillin ± tazobactam (PTZ), 3GCs or other molecule (4GCs, carbapenems, quinolones, cotrimoxazole; control group), with treatment success at day-7. Recurrence of infection was collected as a secondary outcome, and analyzed accounting for the competing risk of death.

**Results:**

From February 2021 to June 2022, 274 patients were included. *Enterobacter cloacae* was the most prevalent specie (31%). Seventy-eight patients (28%) had PTZ as definitive AMT while 44 (16%) had 3GCs and 152 (56%) were classified in the control group. Day-7 success rate was similar between the 3 groups (74% vs. 73% vs. 68% respectively, *p *= 0.814). Recurrence probability at day-28 was 31% (95% CI 21–42), 40% (95% CI 26–55) and 21% (95% CI 15–28) for PTZ, 3GCs and control groups (*p *= 0.020). In multivariable analysis, choice of definitive AMT was not associated with clinical success, but definitive AMT with 3GCs was associated with recurrence at day-28 [csHR(95%CI) 10.9 (1.92–61.91)].

**Conclusion:**

Choice of definitive antimicrobial therapy was not associated with treatment success at day 7. However, recurrence of pneumonia at day-28 was higher in patients treated with third generation cephalosporins with no differences in mortality or mechanical ventilation duration.

**Supplementary Information:**

The online version contains supplementary material available at 10.1186/s13054-024-04820-7.

## Background

Ambler class C serine AmpC β-lactamase [1, 2] are produced by a number of Enterobabacterales (AE) and glucose non-fermenting organisms [3]. AmpC confers natural resistance to aminopenicillins and first-generation cephalosporin [3, 4]. Its production generally occurs by one of the three following mechanisms: inducible chromosomal resistance, stable chromosomal de-repression, or via plasmid-mediated ampC genes [2, 5, 6]. In case of stable chromosomal de-repression or plasmid-mediated ampC genes, AmpC production is generally constitutive rather than induced and isolates are expected to test non-susceptible to ceftriaxone and ceftazidime [4]. However, increased AmpC enzyme production resulting from inducible ampC expression can occur in the presence of specific antibiotics (i.e. amoxicillin, clavulanate, cefazolin and imipenem) and results in ceftriaxone and ceftazidime resistance [6–8], despite initially tested as susceptible to these antibiotics. This risk seems to differ according to the AE species and is mostly described for *Enterobacter cloacae*, *Klebsiella aerogenes* and *Citrobacter freundii* and could be associated with poor outcome [9, 10].

Accordingly, some authors recommend the use of cefepime for AE infections at moderate to high-risk of clinically significant AmpC production due to an inducible ampC gene [10], and against the use of third generation cephalosporins (3GCs) or piperacillin ± tazobactam (PTZ), while the risk of emergence of 3GCs resistance seems to be low [4]. In consequence, the use of large spectrum β-lactams in wild-type AE (wtAE) infections is growing with high risk of emergence of resistances [11].

Our objectives were to assess the effect of definitive antimicrobial therapy (AMT) with 3GCs or PTZ on treatment success at day 7 and the risk of recurrence of pneumonia of these strategies in intensive care units (ICU) patients with ventilator-associated pneumonia (VAP) due to wtAE.

## Materials and methods

### Design

We conducted a prospective multicenter observational study in twenty medical and surgical ICUs in France. Consecutive adult patients, admitted to the ICU and receiving invasive mechanical ventilation for at least 48h, diagnosed with an AmpC-producing wtAE VAP and treated with adequate empirical antimicrobial therapy between February 2020 and June 2021 were included. Adequate empirical AMT was considered as adequate if the molecule was tested sensitive on the microbiological susceptibility results. Infections caused by microorganisms with AmpC overproduction or ESBL production, and other non-wtAE, were not included in this study. Patients with inadequate empirical AMT were excluded.

VAP was defined with the help of the CPIS score by a new and persistent infiltrate on chest radiography in a patient ventilated for at least 48 h, associated with at least one of the following [12]: (1) fever (central temperature ≥ 38.3 °C) or hypothermia (< 36.0 °C); (2) leukocytosis (> 10,000 WBC/mm^3^) or leukopenia (≤ 4000 WBC/mm^3^); (3) increase in volume or new onset of purulent sputum; for patients experiencing acute respiratory distress syndrome or other pre-existing/persisting pulmonary infiltrates for whom it was difficult to demonstrate deterioration of the radiologic images, at least one of the three preceding criteria sufficed for inclusion; and (4) positive quantitative cultures of pulmonary secretion samples (> 10^3^ for distal protected aspirate, > 10^4^ for bronchoalveolar lavage, > 10^6^ for tracheal aspiration). WtAE infection refers to an infection for which the microbiological sample grows to wtAE above the retained threshold, whether the culture was mono- or poly-microbial. Appropriate β-lactam therapy was defined as the use of a β-lactam to which the strain was susceptible. Susceptibility was defined according to the European Committee on Antimicrobial Susceptibility Testing (EUCAST) recommendations [13, 14]. AE species included *Enterobacter cloacae, Klebsiella aerogenes, Serratia marcescens, Citrobacter freundii, Providencia spp., Hafnia alvei, and Morganella morganii*. AE were considered “wild type” if they displayed a low-level expression of AmpC enzymes [13].

The study was approved by the Ethical Committee of the French Society of Intensive Care (CE SRLF 21-05) and the National Commission for Informatics and Liberties (2222,145 v 0). The study was not funded and participation was voluntary. Informed consent was obtained with the patient or its relatives. The study was performed in accordance with the ethical standards as laid down in the 1964 Declaration of Helsinki and its later amendments.

### Exposure of interest

The exposure of interest was definitive AMT with PTZ or 3GCs, after susceptibility test results were obtained [14] compared to the use of antibiotics having stable activity against AmpC [4, 15–19], i.e. quinolones, cotrimoxazole, carbapenems or 4GCs control).

### Endpoints

The primary endpoint was treatment success at day 7. Treatment success was defined in a patient alive at day 7 from AMT introduction, as the disappearance of signs and symptoms of infection without a switch of AMT for a broader-spectrum antibiotic, and without a new positive respiratory tract bacteriological sample, during this period [19]. Otherwise, treatment was considered as a failure. Empirical AMT was defined as the treatment administered before the results of the susceptibility pattern. Definitive antimicrobial therapy was the treatment administered for at least 50% of the total treatment course [20, 21]. Definitive AMT could be the same AMT as the empirical treatment in case of no change in molecule. In case of switch, if the molecule was used for more than 50% of the total treatment course, the switched molecule was considered as definitive AMT. Otherwise, it was the first molecule.

Secondary endpoints included recurrence of VAP, new AmpC-producing AE infection, *Clostrioides difficile* infection as previously defined [22], colonization or infection with multi-drug resistant (MDR) bacteria, duration of mechanical ventilation, death at day 28 and in hospital mortality and ICU length of stay.

Microbiologically documented pneumonia recurrence was diagnosed when clinical signs reappeared within the 28 first days after AMT start, and at least one bacterial species grew at a significant concentration from samples collected. Recurrence was considered as a relapse if the initial causative bacterial strain (i.e., same genus and species) grew at a significant concentration from a second distal sample; it was considered a superinfection otherwise [23].

Multi-drug resistance was defined as a pathogen producing extended-spectrum beta-lactamase (ESBL) or carbapenemase, *Stenotrophomonas maltophilia*, methicillin-resistant *Staphylococcus aureus*, vancomycin-resistant *Enterococcus sp*., or a pathogen resistant to 3 or more antimicrobial classes in accordance with the publication of Magiorakos et al. [24].

### Laboratory methods

Clinical samples were processed and cultured according to the French Microbiology Society recommendations [13, 14]. The susceptibility results to the tested antibiotics were obtained by comparing the minimal inhibitory concentration or the measured inhibition zone diameters to the CASFM–EUCAST clinical breakpoints. Strains categorized as sensitive to cefotaxime and ceftazidime were considered to have a basal level of AmpC.

### Data collection

Data were submitted through an electronic data capture platform (REDCAP™) [25]. Baseline characteristics data were collected and included age; gender; co-morbidities and the Charlson comorbidity index [26]; admission category and diagnosis. The severity of illness was evaluated using the Simplified Acute Physiology Score (SAPS) II [27] on ICU admission and the Sequential Organ Failure Assessment (SOFA) [28] score at ICU admission, antibiotics initiation day and 3 days after AMT initiation. The presence of multi-drug-resistant MDR pathogens [24] at ICU admission and/or detected before day 2, or the emergence of MDR pathogens (i.e., MDR pathogens detected between day 2 and day 28 and not present before) were collected. The need for supportive therapy, number of days in the ICU and hospital, ICU and hospital mortality were also recorded.

Infection and antimicrobial treatment -related data included: type of sample, causative pathogens and susceptibility patterns, type and timing of all antimicrobial agents that were initiated and stopped and dosing (for dosing schemes in case of normal renal function, see Additional file 1: Table S1).

### Statistical analysis

Continuous variables, expressed as median (1st–3rd quartiles), were compared using the Mann–Whitney U or the Kruskall-Wallis test, as appropriate. Categorical variables, expressed as frequencies (percentages), were compared with χ2 or Fisher exact tests, as needed.

To analyze the relationship between definitive AMT and day-7 clinical success, we used a two-steps approach. We first used a mixed logistic regression to account for the center effect. Clinically relevant variables presumed to be associated with the outcome were used to create a final multivariable model namely: age[29], sex[30], body mass index [31], SAPS 2 [27], SOFA score at AMT start [28], CPIS [12], high risk of AmpC over-expression specie [10], total AMT duration[23], definitive AMT and the use of carbapenem or cefepime for empiric AMT (to take account of any inoculum reduction before the switch of AMT). To ensure the robustness of the results, we then performed sensitivity analysis using propensity score (PS) for the probability of definitive AMT with PTZ or 3GCs. Covariates presumed to be associated with the outcome and the choice of definitive treatment were included, namely: age, sex, the Charlson Comorbidity Index, CPIS score, respiratory SOFA at AMT start, SARS-CoV-2 pneumonia [32], high risk of AmpC overexpression bacteria and empiric AMT with Carbapenem or Cefepime. A detailed description of PS analysis is provided in the Additional files 2, 3: Tables S2, S3; Additional file 4: Fig. S1.

The risk of recurrence of VAP at day 28 after AMT start was assessed using a competitive risk approach. Indeed, the outcome is impacted by the time of onset of the event of interest. Patients who had not experienced the event at the end of follow-up (day 28) were censored. Death (from any causes) before recurrence of VAP was a competing event. For that purpose, outcome was assessed using a competing risk model (Gray test, and cumulative incidence curves). To adjust for confounders, we used a Cox cause-specific model regression to create a final multivariate model considering the type of definitive treatment, the risk of AmpC overexpression, the total AMT duration.

All statistical tests were two-sided, with *p *≤ 0.05 considered significant. Statistical analysis was computed with R software (Version 3.6.3; R Foundation for Statistical Computing, Vienna, Austria) and using MatchIt, crmpsk, twang and gbm packages, notably.

## Results

From February 2020 to June 2021, a total of 283 patients with VAP with wtAE were included. Three patients were excluded because AE was considered resistant to piperacillin. Six more patients were excluded from the analysis as the initial AMT was either inappropriate (*n *= 2) or missing (*n *= 4). Thus, 274 patients were finally included in the current analysis.

Baseline characteristics of the patients included in the study are given in Table 1. Briefly, patients were mostly male (77%), median age was 61 [51;70] years. Median SAPS II and SOFA at ICU admission were, respectively, 42 [30;60] and 4 [2;11].

**Table 1:** Characteristics of the patients at baseline

Variable	All patients *N *= 274	Control group *N *= 152	3GCs-definitive AMT *N *= 44	PTZ-definitive AMT *N *= 78	*p* value
Age (years)	61.0 [51;70]	61 [53;70]	60 [50;70]	62 [50;69]	0.782
Sex (male), *n* (%)	210 (76.6)	112 (73.7)	37 (84.1)	61 (78.2)	0.331
Body mass index (kg/m^2^)	28.4 [24.1;32.7]	29.0 [24.0;32.7]	27.2 [24.7;30.9]	28.7 [23.5;34.0]	0.857
Charlson	1 [0;2]	1 [0;1]	0 [0;1]	1 [0;2]	0.185
Immunosuppression, *n* (%)	38 (13.9)	19 (12.5)	6 (13.6)	13 (16.7)	0.687
SAPS II	42 [30;60]	40 [31;58]	47 [29;66]	45 [30;61]	0.622
SOFA at admission	4 [2;11]	4 [2;10]	6 [3;13]	4 [2;12]	0.268
Reason of invasive mechanical ventilation *n *(%)					0.273
Acute respiratory failure	182 (66.4)	111 (73)	23 (52.3)	48 (61.5)	
Coma	36 (13.1)	18 (11.8)	8 (15.9)	11 (14.1)	
Circulatory failure	11 (4)	4 (2.6)	3 (6.82)	4 (5.13)	
Surgery or trauma	12 (4.4)	5 (3.29)	4 (9.09)	3 (3.85)	
Other	33 (12)	14 (9.2)	7 (15.9)	12 (15.4)	
COVID-19 pneumonia *n* (%)	148 (54)	88 (57.9)	19 (43.2)	41 (52.6)	0.273
MDR pathogens at admission *n* (%)	11 (4.01)	8 (5.26)	0 (0)	3 (3.85)	0.321

Categorical variables are expressed as number (%) and continuous variables as median [interquartile ranges]

*SAPS* Simplified acute physiology score, *SOFA* Sequential organ failure assessment, *COVID* Coronavirus infectious disease, *MDR* Multi-drug resistant, *AMT* Antimicrobial therapy, *3GCS* Third-generation cephalosporins, *PTZ* Piperacillin ± tazobactam

Among the 274 patients, 78 (28%), 44 (16%) and 152 (56%) were in the PTZ, 3GCs and control groups, respectively. No difference in baseline characteristics was found between the 3 groups.

### Infection and treatment characteristics

**Fig. 1:**
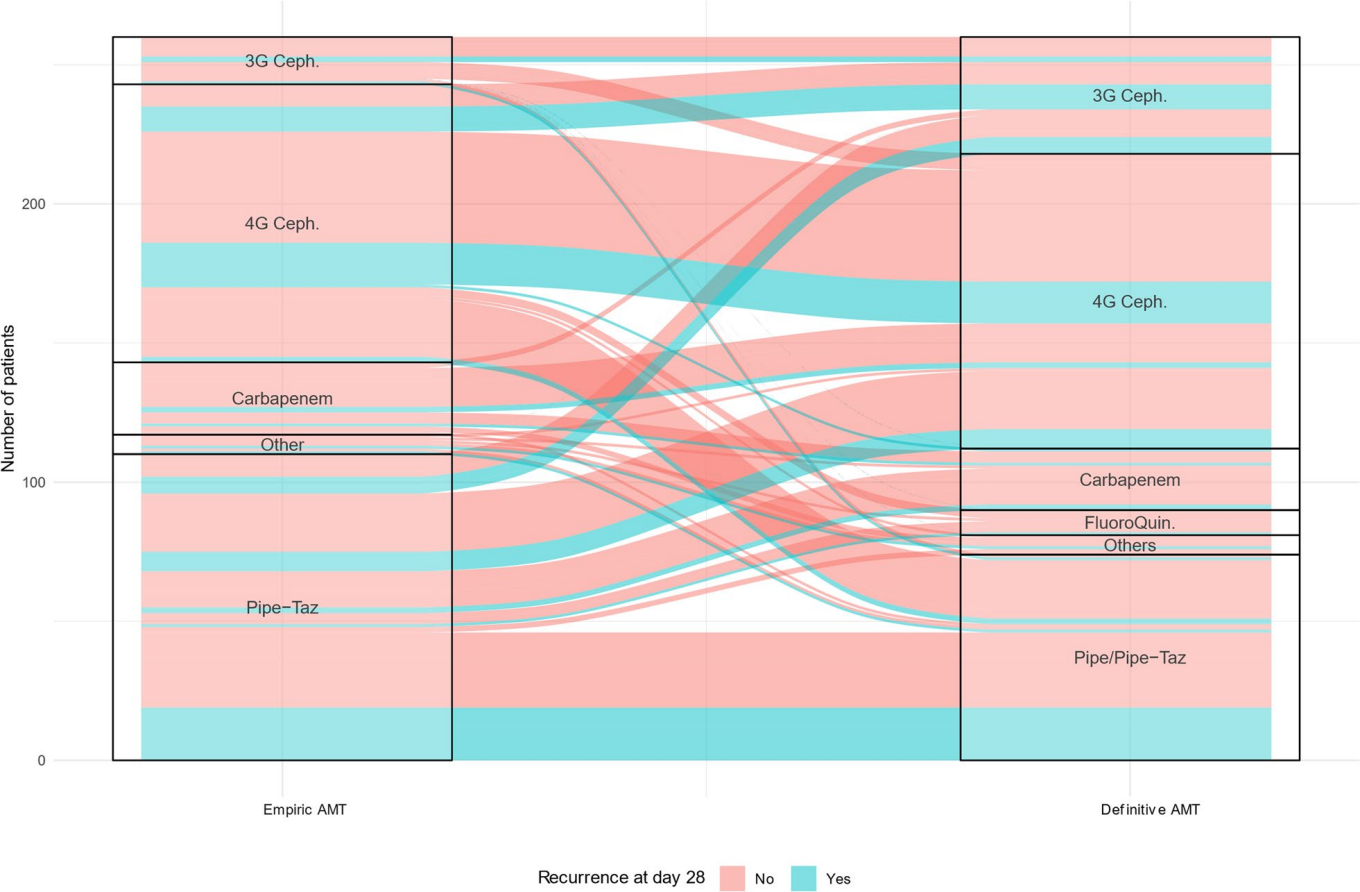
Alluvia plot representing antimicrobial therapy course during first ventilator associated pneumoniae due to wild-type enterobacterale. 3GCeph: third generation cephalosporin; 4GC: fourth generation cephalosporin; Pipe: piperacillin; Taz: tazobactam; AMT: antimicrobial therapy

The first wtAE VAP occurred 7 [4;12] days after MV initiation (Table 2). At AMT start, the median SOFA was 7 [5; 10]. The most frequently identified wtAE was *Enterobacter cloacae* (31%). Definitive AMT, when it differed from empirical AMT, was started 2 [2;4] days after AMT start. AMT course for the first episode of VAP is given in Fig. 1**.** Total length of antimicrobial treatment was 6 [6;7], 6 [5;7], and 7 [6;8] days in the PTZ, 3GCs and control group, respectively (*p *= 0.001).

**Table 2:** Characteristics and treatment of the first wild-type AmpC-producing enterobacterales ventilator associated pneumoniae

Variable	All patients *N *= 274	Control group *N *= 152	3GCs-definitive AMT *N *= 44	PTZ-definitive AMT *N *= 78	*p* value
*Clinical characteristics*					
Time from MV to first wtAE VAP (days)	7 [4;12]	8 [5;15]	6 [5;9]	8 [4;11]	0.256
SOFA at AMT start	7 [5;10]	7 [6;10]	7 [4;10]	9 [6;12]	0.152
Respiratory score	3 [2;3]	3 [3;4]	3 [2;3]	3 [2;4]	0.133
Cardiovascular score	3 [0;4]	3 [0;4]	0 [0;3]	3 [0;4]	0.254
Renal score	0 [0;0]	0 [0;0]	0 [0;0]	0 [0;0]	0.946
ECMO support (*n*%)					0.448
VV	35 (12.5)	19 (12.6)	3 (6.8)	12 (15.6)	
VA	6 (2.2)	2 (1.3)	1 (2.3)	3 (3.9)	
CPIS	5 [4;7]	6 [4;7]	6 [4;7]	5 [4;6]	0.749
*Microbiological characteristics*					
Isolated AmpC-producing enterobacterales					0.016
*Enterobacter cloacae*	84 (30.7)	54 (35.5)	4 (9.09)	26 (33.3)	
*Citrobacter freundii*	10 (3.65)	3 (1.9)	3 (6.8)	4 (5.1)	
*Klebsiella aerogenes*	69 (25.2)	32 (21.1)	20 (45.5)	17 (21.8)	
*Hafnia alvei*	31 (11.3)	14 (9.2)	6 (13.6)	11 (14.1)	
*Morganella morganii*	14 (5.1)	10 (6.6)	2 (4.6)	2 (2.6)	
*Serratia marcescens*	60 (21.9)	36 (23.7)	9 (20.5)	15 (19.2)	
*Other*	6 (2.2)	3 (1.9)	0 (0)	3 (3.9)	
Polymicrobial infection	134 (49.3)	80 (53)	21 (47.7)	33 (42.9)	0.343
*AMT of VAP*					
Combination empirical AMT	61 (22.3)	31 (20.4)	5 (11.4)	25 (32.1)	0.022
Empirical AMT with cefepime or carbapenem	135 (49.3)	90 (59.2)	20 (45.5)	25 (32.1)	< 0.001
SOFA at day 3 after AMT start	7 [3;9]	7 [3;9]	5 [2;8]	7 [4;11]	0.161
Total duration of AMT (days)	7 [6;7]	6 [6;7]	6 [5;7]	7 [6;8]	0.001

Categorical variables are expressed as number (%) and continuous variables as median [interquartile ranges]

*ICU* Intensive care unit, *wtAE* Wild-type AmpC-producing enterobacterales, *MV* Mechanical ventilation, *VAP* Ventilator associated pneumoniae, *AMT* Antimicrobial treatment, *SOFA* Sequential organ failure assessment, *CPIS* clinical pulmonary infection score, *ECMO* Extracorporeal membrane oxygenation, *VV* Veno-venous, *VA* Veno-arterial, *3GCs* Third-generation cephalosporins, *PTZ* Piperacillin ± tazobactam

### Primary endpoint

Clinical success at day-7 occurred in 184 (69%) of cases with no difference between the 3 groups (*p *= 0.814, Table 3). In multivariable analysis, definitive AMT was not associated with the primary outcome [1.07 (0.56–2.08) and 0.93 (0.41–2.10) for PTZ and 3GCs categories compared to the control group, respectively (Additional file 2: Table S2). Propensity score analysis yielded similar results (Additional file 3: Table S3).

**Table 3:** Outcome of patients according to definitive antimicrobial therapy

Variable	Control group *N *= 152	3GCs-definitive AMT *N *= 44	PTZ-definitive AMT *N *= 78	*p* value
Treatment success at day 7 *n* (%)	100 (68)	32 (72.7)	52 (74.4)	0.814
Recurrence of VAP at day 28 n (%)				0.054
Relapse	15 (10.4)	12 (28.6)	13 (16.6)	
Superinfection	15 (10.4)	5 (11.9)	9 (11.5)	
Death at day 28 *n* (%)	28 (18.5)	8 (18.2)	14 (17.9)	0.997
Death during ICU stay *n* (%)	53 (35.3)	12 (27.3)	20 (25.6)	0.311
Death during hospital stay *n* (%)	55 (36.2)	14 (31.8)	21 (27.6)	0.425
Duration of MV (days)	25 [13;41]	18 [11;33]	17[12;31]	0.092

Categorical variables are expressed as number (%) and continuous variables as median [interquartile ranges]

*VAP* Ventilator associated pneumonia, *ICU* Intensive care unit, *MV* mechanical ventilation, *3GCs* third-generation cephalosporins, *3GCs* 3GCS used as definitive treatment, *PTZ* piperacillin ± tazobactam used as definitive treatment

### Secondary endpoints

Vital status at day 28, ICU and hospital discharge were similar between the 3 groups (Table 3)**.** Recurrence occurred in 28, 39 and 20% of patients in the PTZ, 3GCs and control group, respectively (*p *= 0.054). To note, in the PTZ group, recurrence occurred in only 2 (8%) patients among those treated with carbapenems or cefepime as empirical AMT (Fig. 1). AmpC overproduction occurred in 30% of relapses and was not associated with definitive treatment group (*p *= 0.056). Number of days alive without AMT at day 28 was no different between the three groups (10 [0;20], 10 [0;20] and 12 [2;21] days for control, 3GCs and PTZ groups, respectively, *p *= 0.099).

**Fig. 2:**
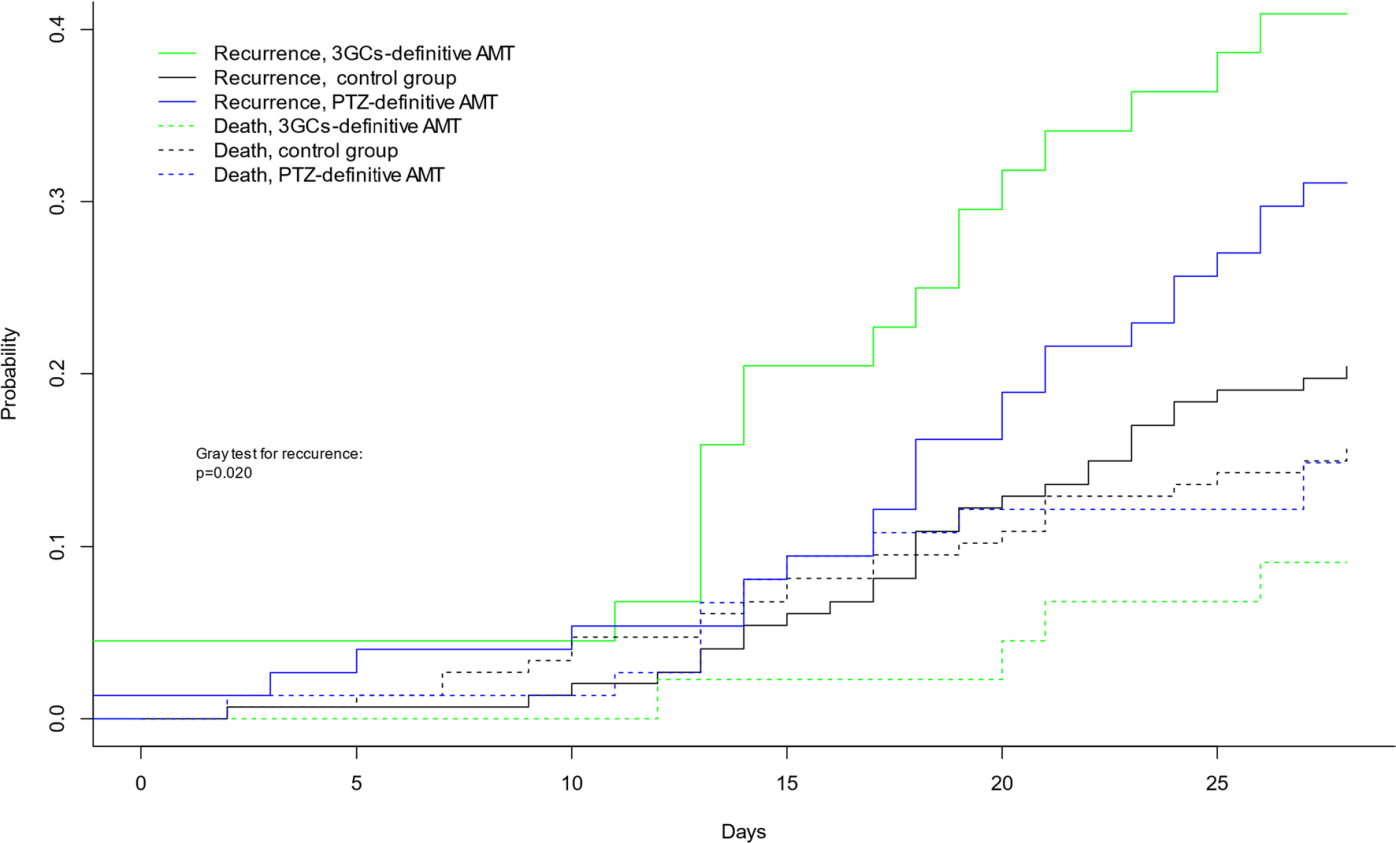
Cumulative incidence function (CIF) curves of recurrence and death according to the treatment of first ventilator associated pneumonia due to wild-type AmpC producing Enterobacterale. AMT: antimicrobial therapy; 3GCS: third-generation cephalosporins; 3GCs definitive AMT: 3GCS used as definitive treatment; PTZ definitive AMT: piperacillin ± tazobactam used as definitive treatment. CIF of mortality were not statistically different (gray test *p *= 0.60)

Accounting for the competing risk of death, the probability of recurrence at day 28 was 31% (95%CI 21–42), 40% (95%CI 26–55) and 21% (95%CI 15–28) in the PTZ, 3GCs and control groups, respectively (Fig. 2, *p *= 0.020). Similar results were obtained when only relapse was considered (Additional file 5: Fig. S2). In multivariable analysis, 3GCs was associated with recurrence [csHR(95%CI) 10.9 (1.92–61.91), *p *= 0.006], as well as high risk of AmpC overexpression AE species [csHR(95%CI) 1.82 (1.13–2.96), Additional file 3: Table S3].

Forty-six patients (17%) acquired MDR bacteria with no difference between groups, and only 5 (2%) had *Clostrioides difficile* colitis.

## Discussion

In this prospective multicenter cohort including 274 patients with VAP due to AmpC producing wtAE, we found that: (1) definitive AMT with PTZ or 3GCs was not associated with clinical treatment failure at day-7; (2) this strategy might be associated with a higher risk of recurrence (including relapse at day 28), especially with 3GCs definitive treatment; (3) the incidence of MDR bacteria was not lower in patients with definitive treatment by piperacillin or 3GCs.

The lack of significant association between de-escalation and clinical success remains a matter of debate regards to conflicting results recently published. Similarly to our results, the primary endpoint of the MERINO2 trial (composite of death, clinical or microbiological failure, and microbiological relapse) did not significantly differ between PTZ and meropenem, while the risk of microbiological failure was higher in the PTZ group (13 vs. 0%) [33]. In the other hand, Mounier et al. observed a treatment failure rate of 29% in a cohort of 177 critically ill patients. The authors also found that definitive treatment with cefotaxime was associated with a better outcome. These findings are in conflict with ours as well as with others recently published in which both the risk of therapeutic failure (32% vs. 18%) and of emergence of AmpC overproducing strains (13% vs. 5%) were increased in patients treated by 3GCs compared to PTZ [34]. Furthermore, definitive treatment with PTZ or cefotaxime was associated with a higher rate of treatment failure [HR 95% CI 3.13 (1.69–5.80) and 6.81 (3.76–12.4), respectively] in bloodstream infection or pneumonia in a cohort of 575 patients [35]. In this retrospective study, treatment failure was however defined as a composite outcome including death at day 30, recurrence and breakthrough infection, and may be difficult to interpret regarding the risk of clinical failure. Overall, it seems difficult to compare all these recently published findings as those studies included patients with different infections of different severity. Our findings apply whatsoever only on critically ill patients treated for VAP.

In the present study, considering the competing risk of death, recurrence happened more frequently in patients with 3GCs definitive treatment (39%) and PTZ (28%) compared to control group (20%). This is consistent with recent studies [7, 21, 34, 35], which found that the risk especially existed in critically ill patients undergoing mechanical ventilation, and increased in the case of *Citrobacter freundii, Klebsiella aerogenes and Enterobacter cloacae* documented infection. These species are known to be at high risk of AmpC overexpression [36] and the usually accepted management of such infections included cefepime of carbapenems [10, 36]. However, definitive treatment by 3GCs or PTZ may still be possible in other species [10, 36], especially after empiric AMT with cefepime or carbapenem. This possibility is however still debated [4, 35]. In the present study, among the PTZ patients, only 8% on those treated with carbapenems or cefepime as empiric AMT presented recurrence at day 28. This very low rate of recurrence may be explained by an inoculum effect [37]. Indeed, some studies suggest a dramatic increase in β-lactam MICs with high inoculum [38]. Once that inoculum has been reduced with a molecule with low affinity for the AmpC β-lactamase such as cefepime, the risk of selecting a clone with AmpC overexpression seems to be small [4], as well as the risk of relapse [21, 38].

We acknowledge several limitations. First, we included only VAP and our results cannot be extended to other type of infections. However, wtAE VAP is frequent in ICU patients and may be the main site of wtAE infection [21]. Second, due to the observational design of our study, we cannot exclude the persistence of selection biases with, in particular, a center effect in the decision to switch the initial AMT, as we did not collect the reason of the choice of the definitive treatment. However, we tried to take into account this issue using a mixed effect regression [39]. Third, we did not collect serum antibiotic levels and cannot rule out a suboptimal dosage in some patients, particularly those under ECMO. However, in the great majority of cases, physicians used high-dose regimens and the risk is thus pretty low. Fourth, while the design is only observational, we did not have control of empirical AMT, and outcomes may differ according to the choice of the molecule. We tried to limit this bias with the PS analysis, which included this variable (i.e. empirical AMT with carbapenem or cefepime), but uncertainty remains considering the risk of relapse, and only a randomized control trial with AMT standardization can eliminate this confounding factor. Fifth, no adjudication committee was available for this study, and the assessment of the outcomes depended of the investigator with a risk of evaluation bias, because the diagnosis of failure or recurrence necessarily depends on the attitude of the attending physician and one may consider, with a new positive culture, that there is sufficient clinical and biological argument for the diagnosis of failure/recurrence of pneumonia and modify the AMT, while another may not. However, it reflects real life and we tried to use the most objective criteria available (death or new positive biological sample and clinical signs leading to new AMT, strengthening the diagnosis of failure or recurrence). We expect that the attitude is the same for the patients included in one center, and the bias limited by our statistical approach. Finally, more than 50% of the patients included in the cohort were mechanically ventilated for COVID-19, which limits the generalizability of our results, as these patients may have a higher rate of recurrence of pneumonia.

## Conclusion

In conclusion, this study showed that antibiotic definitive treatment of VAP due to wtAE with piperacillin ± tazobactam or 3GCs was not associated with treatment failure at day 7. However, recurrence of pneumonia at day 28 was higher in patients with definitive treatment 3GCs without difference in mortality.

### Supplementary Information

**Additional file 1. Supplementary Table 1.** Dosing schemes of intravenous antibiotics in case of normal renal function.

**Additional file 2.** **Supplementary Table 2.** Multivariable analysis with mixed effect regression for primary outcome.

**Additional file 3.** **Supplementary Table 3.** Multivariable analysis for primary and secondary outcomes.

**Additional file 4. Supplemental Figure 1.** Detailed description of propensity score (PS) analysis. **Panel A.** Distribution of the PS according to the treatment (Tx) group. **Panel B.** Balance of the covariates in treatment groups before and after weighting sample using inverse probability treatment weighting.

**Additional file 5. Supplemental Figure 2.** Cumulative incidence function (CIF) curves of relapse and death according to the treatment of first ventilator associated pneumonia due to wild type AmpC producing Enterobacterale. AMT: antimicrobial therapy; 3GCS: third-generation cephalosporins; PTZ: piperacillin +/- tazobactam used as definitive treatment.

## Data Availability

The datasets used and analyzed during the current study are available from the corresponding author on reasonable request.

## Abbreviations

3GCs: Third generation cephalosporins
4GCs: Fourth generation cephalosporins
AMT: Antimicrobial therapy
CPIS: Clinical pulmonary infection score
ESBL: Extended-spectrum beta-lactamase (ESBL)
EUCAST: European Committee on Antimicrobial Susceptibility Testing
ICU: Intensive Care Unit
MDR: Multi-drug resistant
PTZ: Piperacillin ± Tazobactam
PS: Propensity score
SAPS II: Simplified acute pulmonary score II
SOFA: Sequential Organ Failure Assessment
VAP: Ventilator associated pneumonia
wtAE: Wild type AmpC-producing enterobacterales
WBC: White blood cells
