# Supplementary material for: Antibiotic definitive treatment in ventilator associated pneumonia caused by AmpC-producing Enterobacterales in critically ill patients: a prospective multicenter observational study
Source: Crit Care. 2024 Feb 5;28:40. doi: 10.1186/s13054-024-04820-7 (PMC10845500; doi:10.1186/s13054-024-04820-7)
Supplement: Supplementary file 1 — Additional file 1. Supplementary Table 1. Dosing schemes of intravenous antibiotics in case of normal renal function. [file 13054_2024_4820_MOESM1_ESM.docx]

**Supplemental Table 1. Dosing schemes of intravenous antibiotics in case of normal renal function.**

| **Molecule** | **Dosing scheme** |
| --- | --- |
| **Meropenem** | 2000mg per 8 hours with a >30 min intermittent infusion |
| **Imipenem/cilastatin** | 1000 mg per 8 to 6 hours with a > 30 min intermittent infusion |
| **Piperacillin** | 4000mg per 6 to 8 hours* with prolonged infusion of 4 hours after a loading dose |
| **Cefepime** | 2000 mg per 8 to 6 hours with prolonged infusion of 4 hours or continuous infusion after loading dose |
| **Ceftriaxone** | 2000 to 3000 mg per 24 hours, intermittent infusion |
| **Cefotaxime** | 1000 ** to 2000 mg every 8 hours with >30 min Intermittent infusion or prolonged infusion of 4 hours or continuous infusion after loading dose |
| **Ceftazidime** | 2000 mg every 8 hours with >30 min Intermittent infusion or prolonged infusion of 4 hours or continuous infusion after loading dose |
| **Levofloxacin** | 500 mg per 12 hours |
| **Ciprofloxacin** | 400 mg per 8 hours |
| **Cotrimoxazole** | 800mg/160mg per 8 hours using 4 hours IV administration |

*** only 9 patients among the cohort (empiric and definitive antimicrobial therapy)**

**** only four patients among the cohort (empiric and definitive antimicrobial therapy)**
