# Supplementary material for: Antibiotic definitive treatment in ventilator associated pneumonia caused by AmpC-producing Enterobacterales in critically ill patients: a prospective multicenter observational study
Source: Crit Care. 2024 Feb 5;28:40. doi: 10.1186/s13054-024-04820-7 (PMC10845500; doi:10.1186/s13054-024-04820-7)
Supplement: Supplementary file 2 — Additional file 2. Supplementary Table 2. Multivariable analysis with mixed effect regression for primary outcome. [file 13054_2024_4820_MOESM2_ESM.docx]

**Supplementary table 2. Multivariable analysis with mixed effect regression for primary outcome.**

| **Variable** | **Odd Ratio**  **(95% CI)** | **p-value** |
| --- | --- | --- |
| Age | 0.98 (0.96-1.00) | 0.082 |
| Male sex | 0.69 (0.33-1.43). | 0.314 |
| Body mass index (kg/m²) | 1.01 (0.97-1.05) | 0.605 |
| SAPS II | 1.01 (0.97-1.05) | 0.217 |
| CPIS score | 1.08 (0.91-1.27) | 0.368 |
| SOFA at AMT start | 0.85 (0.78-0.92) | 0.001 |
| High risk AmpC-producing enterobacterale specie | 0.69 (0.39-1.24) | 0.216 |
| Use of carbapenem or cefepime for empiric AMT | 1.12 (0.62-2.01) | 0.694 |
| Total AMT duration (days) | 0.98 (0.88-1.10) | 0.719 |
| PTZ-definitive AMT | 1.07 (0.56-2.08) | 0.831 |
| 3GCs-definitive AMT | 0.93 (0.41-2.10) | 0.865 |

*CPIS clinical pulmonary infection score, SAPS simplified acute pulmonary score, SOFA sequential organ failure assessment, AMT antimicrobial therapy, 3GCs third-generation cephalosporins, PTZ: piperacillin +/- tazobactam*
