# Supplementary material for: Antibiotic definitive treatment in ventilator associated pneumonia caused by AmpC-producing Enterobacterales in critically ill patients: a prospective multicenter observational study
Source: Crit Care. 2024 Feb 5;28:40. doi: 10.1186/s13054-024-04820-7 (PMC10845500; doi:10.1186/s13054-024-04820-7)
Supplement: Supplementary file 3 — Additional file 3. Supplementary Table 3. Multivariable analysis for primary and secondary outcomes. [file 13054_2024_4820_MOESM3_ESM.docx]

**Supplementary Table 3. Multivariable analysis for primary and secondary outcomes.**

|  | **PTZ-definitive AMT versus control group** | **3GCs-definitive AMT versus control group** |
| --- | --- | --- |
| **Clinical success at day 7** |  |  |
| Mixed effect regression model, OR (95% CI) | 1.07 (0.56-2.08) | 0.93 (0.41-2.10) |
| Propensity score analysis, OR (95% CI) | 1.05 (0.92-1.20) | 1.11 (0.94-1.31) |
| **Recurrence at day 28,** csHR (95%CI), n=68 | 4.34 (0.76-23.9) | 10.9 (1.92-61.91) |
| **Mortality at day-28,** csHR (95%CI), n=37 | 0.45 (0.06-3.40) | 0.02 (0.00-0.28) |

*OR: odd ratio; csHR: cause specific hazard ratio; 3GCs: third-generation cephalosporins; No-ADE: no de-escalation; PTZ: piperacillin +/- tazobactam, AMT: antimicrobial therapy*
