# Supplementary material for: Antibiotic definitive treatment in ventilator associated pneumonia caused by AmpC-producing Enterobacterales in critically ill patients: a prospective multicenter observational study
Source: Crit Care. 2024 Feb 5;28:40. doi: 10.1186/s13054-024-04820-7 (PMC10845500; doi:10.1186/s13054-024-04820-7)

**Supplementary Figure 1. Detailed description of propensity score (PS) analysis**

As a secondary analysis, generalized propensity score methods were used to estimate the effect of de-escalation on patient outcomes. These analyses used the three-level classification of de-escalation described above: control group vs. PTZ-definitive AMT vs. 3GCS-definitive AMT. We used the framework described by McCaffrey et al. when the exposure variable had more than two levels(3). To do so, we conducted a series of three pairwise comparisons (control group vs. PTZ-definitive AMT; control group vs. 3GCs-definitive AMT; PTZ-definitive AMT vs. 3GCs-definitive AMT). For each pairwise comparison, we estimated the effect for those patients who were treated in a de-escalation strategy. Generalized boosting methods were used to estimate the propensity score. Input predictor variables were those measured variables thought to be prognostic of the study outcomes: age, sex, the Charlson Comorbidity Index, CPIS score, respiratory SOFA at AMT start, SARS-CoV-2 pneumonia, high risk of AmpC overexpression bacteria and empiric AMT with Carbapenem or Cefepime). Once the propensity score was estimated for a given pairwise comparison, we computed inverse probability of treatment weights (IPTWs) appropriate for estimating the average treatment effect. The balance in baseline covariates between volume categories was assessed using weighted standardized differences. Standardized differences of less than 0.10 (10%) were taken as indicative of acceptable balance (Panel A and Panel B). Then, in the weighted sample, we estimated the probability of the outcome in each of the two volume categories as the weighted mean of the observed outcomes. We reran the multivariable logistic regression as sensitivity analyses.

**Panel A. Distribution of the PS according to the treatment (Tx) group.**

*AMT: antimicrobial therapy3GCS: third-generation cephalosporins; PTZ: piperacillin+/- tazobactam*


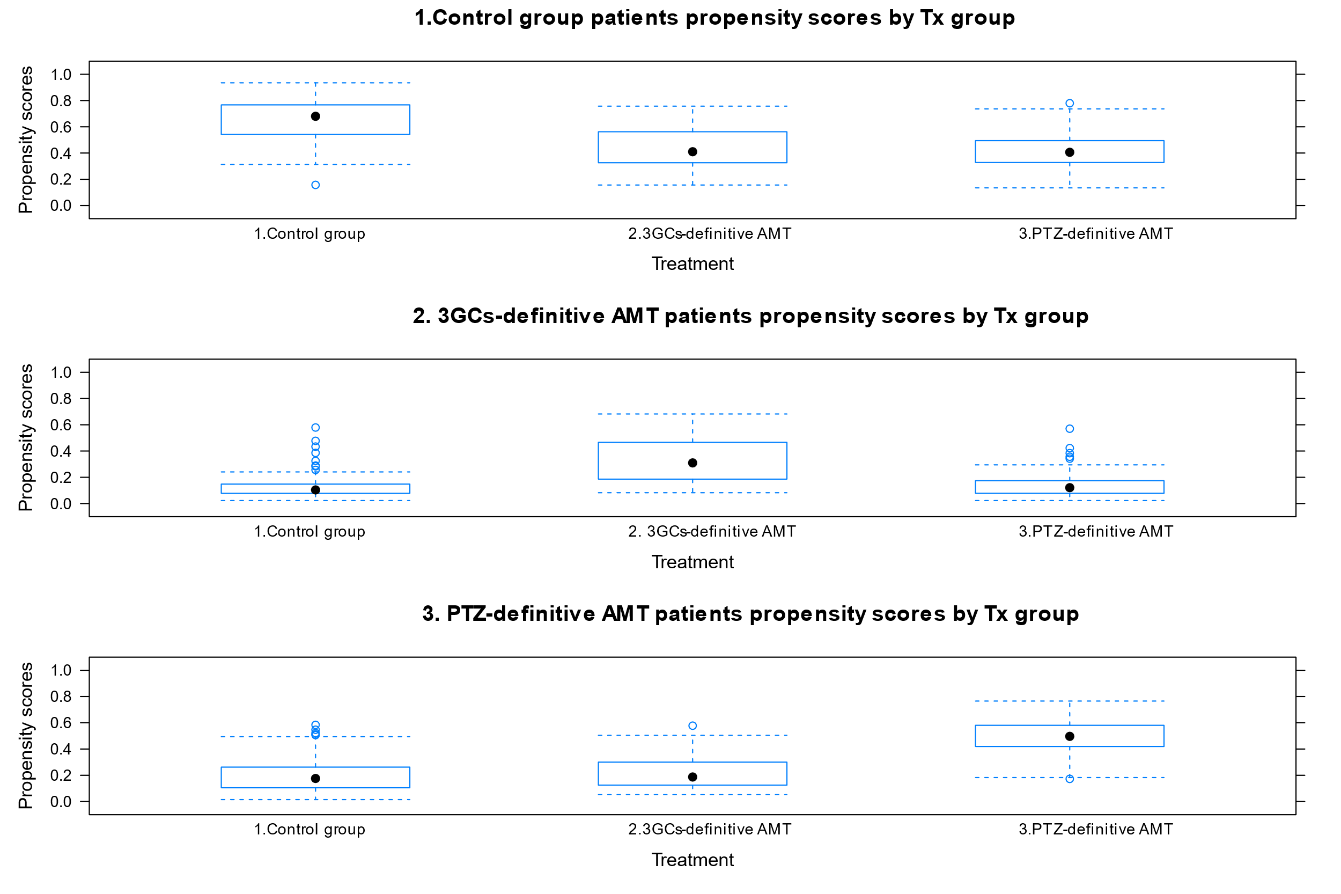


**Panel B. Balance of the covariates in treatment groups before and after weighting sample using inverse probability treatment weighting.**

*AMT: antimicrobial therapy; 3GCS: third-generation cephalosporins; PTZ: piperacillin +/- tazobactam*


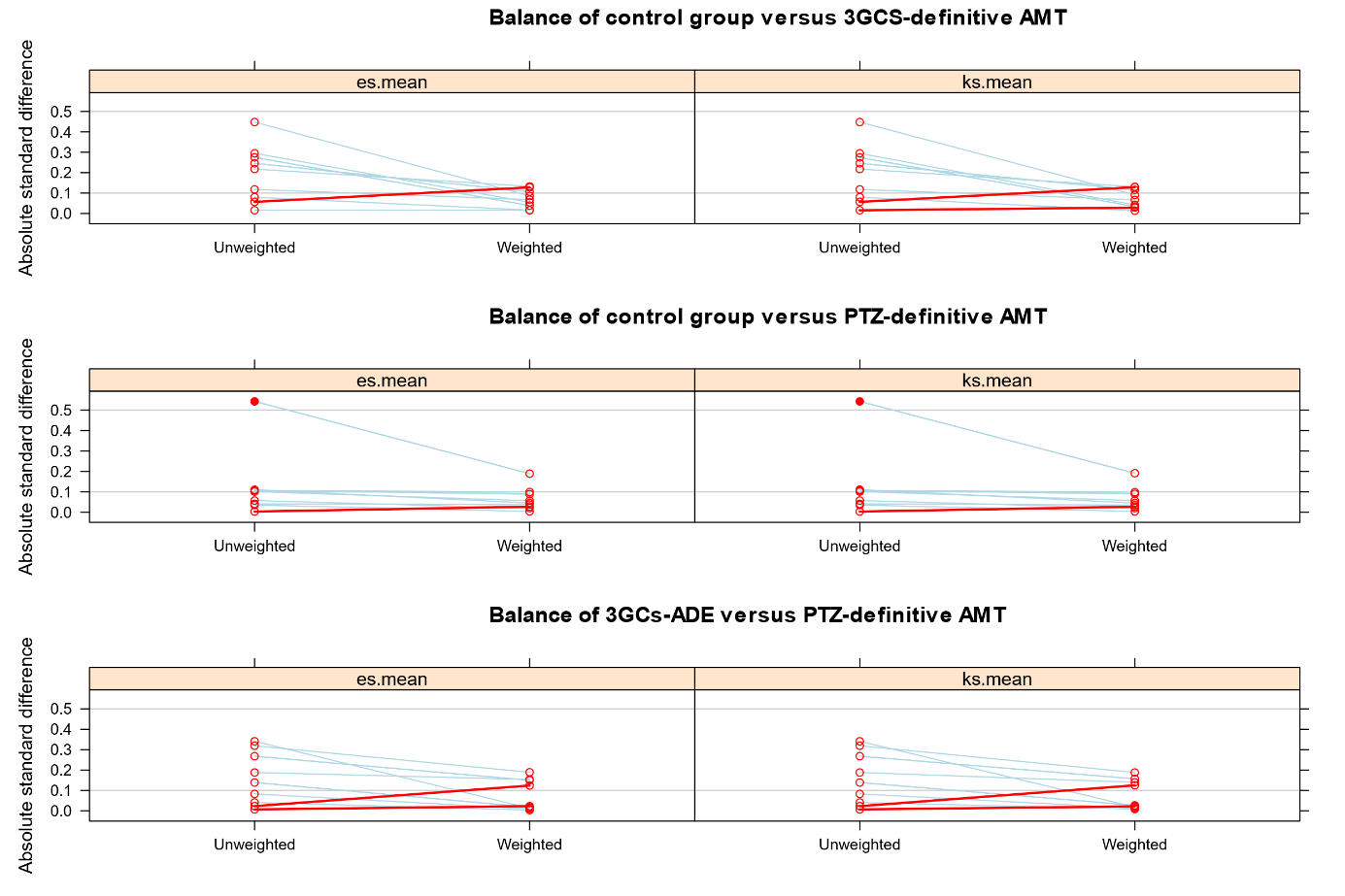

Supplement: Supplementary file 4 — Additional file 4. Supplemental Figure 1. Detailed description of propensity score (PS) analysis. Panel A. Distribution of the PS according to the treatment (Tx) group. Panel B. Balance of the covariates in treatment groups before and after weighting sample using inverse probability treatment weighting. [file 13054_2024_4820_MOESM4_ESM.docx]
