# Supplementary material for: Antibiotic definitive treatment in ventilator associated pneumonia caused by AmpC-producing Enterobacterales in critically ill patients: a prospective multicenter observational study
Source: Crit Care. 2024 Feb 5;28:40. doi: 10.1186/s13054-024-04820-7 (PMC10845500; doi:10.1186/s13054-024-04820-7)
Supplement: Supplementary file 5 — Additional file 5. Supplemental Figure 2. Cumulative incidence function (CIF) curves of relapse and death according to the treatment of first ventilator associated pneumonia due to wild type AmpC producing Enterobacterale. AMT: antimicrobial therapy; 3GCS: third-generation cephalosporins; PTZ: piperacillin +/- tazobactam used as definitive treatment. [file 13054_2024_4820_MOESM5_ESM.docx]

**Supplementary Figure 2. Cumulative incidence function curves of relapse and death after the first ventilator associated pneumonia due to wild type AmpC producing Enterobacterale.**

*AMT: antimicrobial therapy; 3GCS: third-generation cephalosporins; PTZ: piperacillin +/- tazobactam used as definitive treatment*

**
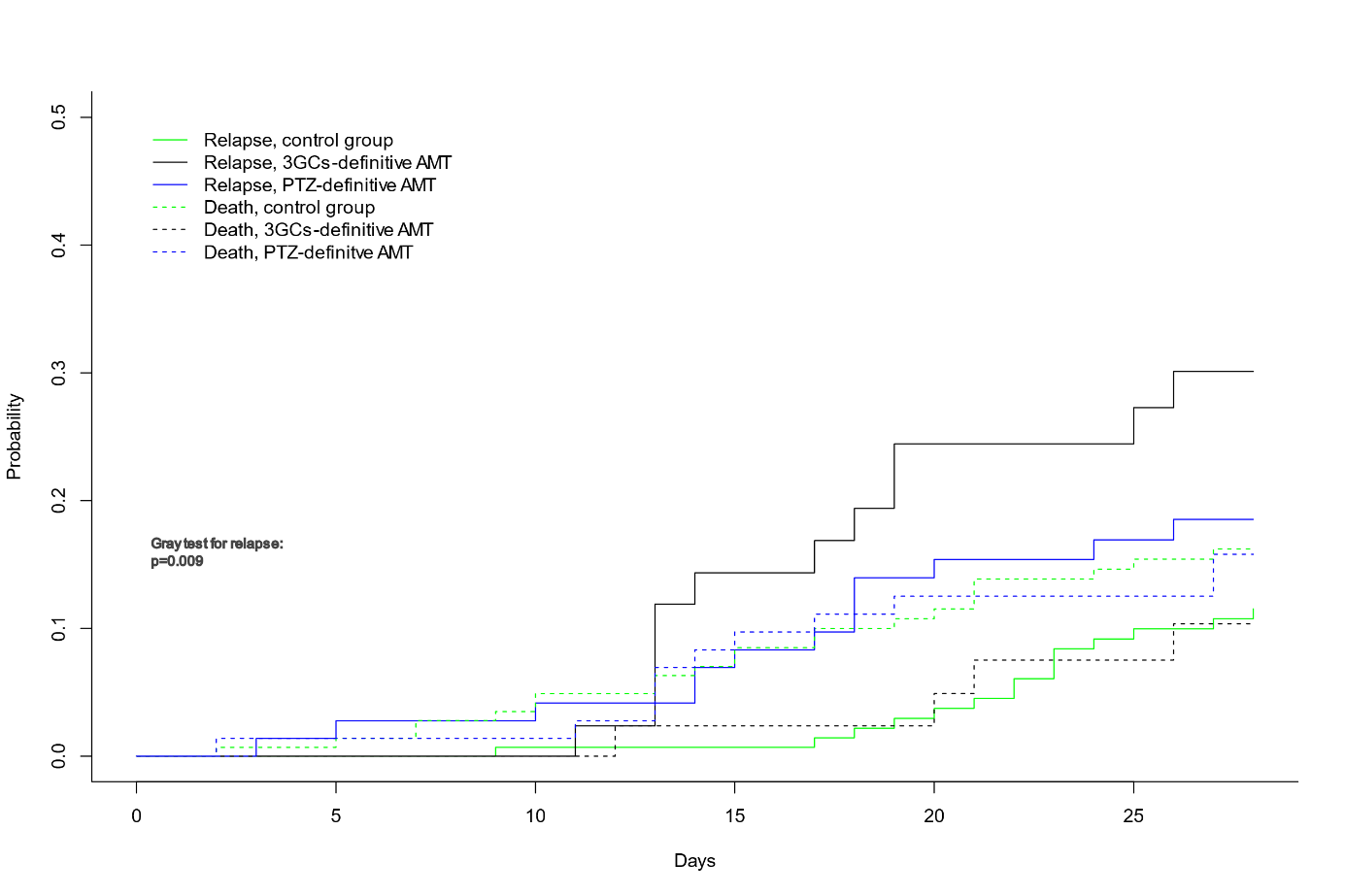
**
